# Dosimetry with a clinical linac adapted to FLASH electron beams

**DOI:** 10.1002/acm2.13270

**Published:** 2021-05-24

**Authors:** Stanislaw Szpala, Vicky Huang, Yingli Zhao, Alastair Kyle, Andrew Minchinton, Tania Karan, Kirpal Kohli

**Affiliations:** ^1^ BC Cancer Surrey Centre Surrey BC Canada; ^2^ BC Cancer Research Centre Vancouver BC Canada; ^3^ BC Cancer Vancouver Centre Vancouver BC Canada

**Keywords:** FLASH, gafchromic film, ionization chamber, ultra‐high dose rate

## Abstract

**Purpose:**

To assess dosimetric properties and identify required updates to commonly used protocols (including use of film and ionization chamber) pertaining to a clinical linac configured into FLASH (ultra‐high dose rate) electron mode.

**Methods:**

An 18MV photon beam of a Varian iX linac was converted to FLASH electron beam by replacing the target and the flattening filter with an electron scattering foil. The dose was prescribed by entering the MUs through the console. Fundamental beam properties, including energy, dose rate, dose reproducibility, field size, and dose rate dependence on the SAD, were examined in preparation for radiobiological experiments. Gafchromic EBT‐XD film was evaluated for usability in measurements at ultra‐high dose rates by comparing the measured dose to the inverse square model. Selected previously reported models of chamber efficiencies were fitted to measurements in a broad range of dose rates.

**Results:**

The performance of the modified linac was found adequate for FLASH radiobiological experiments. With exception of the increase in the dose per MU on increase in the repetition rate, all fundamental beam properties proved to be in line with expectations developed with conventional linacs. The field size followed the theorem of similar triangles. The highest average dose rate (2 × 10^4^ Gy/s) was found next to the internal monitor chamber, with the field size of FWHM = 1.5 cm. Independence of the dose readings on the dose rate (up to 2 × 10^4^ Gy/s) was demonstrated for the EBT‐XD film. A model of recombination in an ionization chamber was identified that provided good agreement with the measured chamber efficiencies for the average dose rates up to at least 2 × 10^3^ Gy/s.

**Conclusion:**

Dosimetric measurements were performed to characterize a linac converted to FLASH dose rates. Gafchromic EBT‐XD film and dose rate‐corrected cc13 ionization chamber were demonstrated usable at FLASH dose rates.

## 1 INTRODUCTION

The FLASH effect is defined as improved normal tissue sparing in radiation therapy when the dose rates are considerably higher (over 40 Gy/s) than those currently employed in clinical practice (about 0.2 Gy/s).^1^ Improved normal tissue sparing in cell lines and in small animals were reported for FLASH radiation therapy.^2^, ^3^, ^4^ Efforts to understand the radiobiological mechanism, including the role of oxygen immediately after a microsecond pulse, are ongoing.^3^, ^5^ Recently, a report on the first patient treated with FLASH radiotherapy was published.^6^

Experimental linear accelerators are available for delivering FLASH dose rates, including Oriatron (PMB‐Alcen, France). Linacs designed for intraoperative radiation therapy, which operate at high dose per pulse sequence, can be also used, for example, Novac 7 (New Radiant Technology, Italy). Recently, clinical linear accelerators were modified to deliver electron beams at FLASH dose rates by tuning nonclinical electron beams (including the pulse forming network voltage and the gun filament current), and by using pulse‐counting circuits to control beam delivery.^7^, ^8^

Dosimetric characterization methodology of FLASH beams was described for modified clinical linacs and experimental linacs.^7^, ^9^ Gafchromic film (EBT2 or EBT3, Ashland, Bridgewater, NJ, USA) was predominantly used in measurements of dose. The independence of Gafchromic EBT and OSL on the dose rate (much higher in FLASH beams than in conventional linacs) was demonstrated through comparing to measurements with a Faraday cup and an integrating current transformer.^10^ Beam current monitoring was used to show similar independence for EBT3 film.^11^ Ionization chambers typically used in conventional linac dosimetry suffer from dose rate dependence at ultra‐high dose rates. This was addressed for parallel‐plate ionization chambers,^12^, ^13^, ^14^, ^15^ through modeling ion recombination using the work of Boag et al.^16^, ^17^

In this paper, we propose a simplified method of controlling a modified clinical Varian linac (Varian Medical Systems, Inc., Palo Alto, CA, USA) in ultra‐high dose rate regime by entering the MUs through the console instead of counting pulses with a microcontroller. We evaluate the dosimetric characteristics of the FLASH electron beam that are important in radiobiological applications. In particular, we investigate dose rate and field size trade‐off at various practically achievable distances from the scattering foil. We demonstrate usefulness of film dosimetry at FLASH rates for EBT‐XD Gafchromic film (less sensitive than other types of Gafchromic film, hence preferred for our high‐dose measurements) by comparing to a model, not to other detectors. We characterize response of a cylindrical chamber at ultra‐high dose rates, and establish a dose rate‐dependent correction by fitting the measured data to previously reported theoretical models.

## 2 METHODS

### 2.A Linac conversion to FLASH

**Fig. 1:**
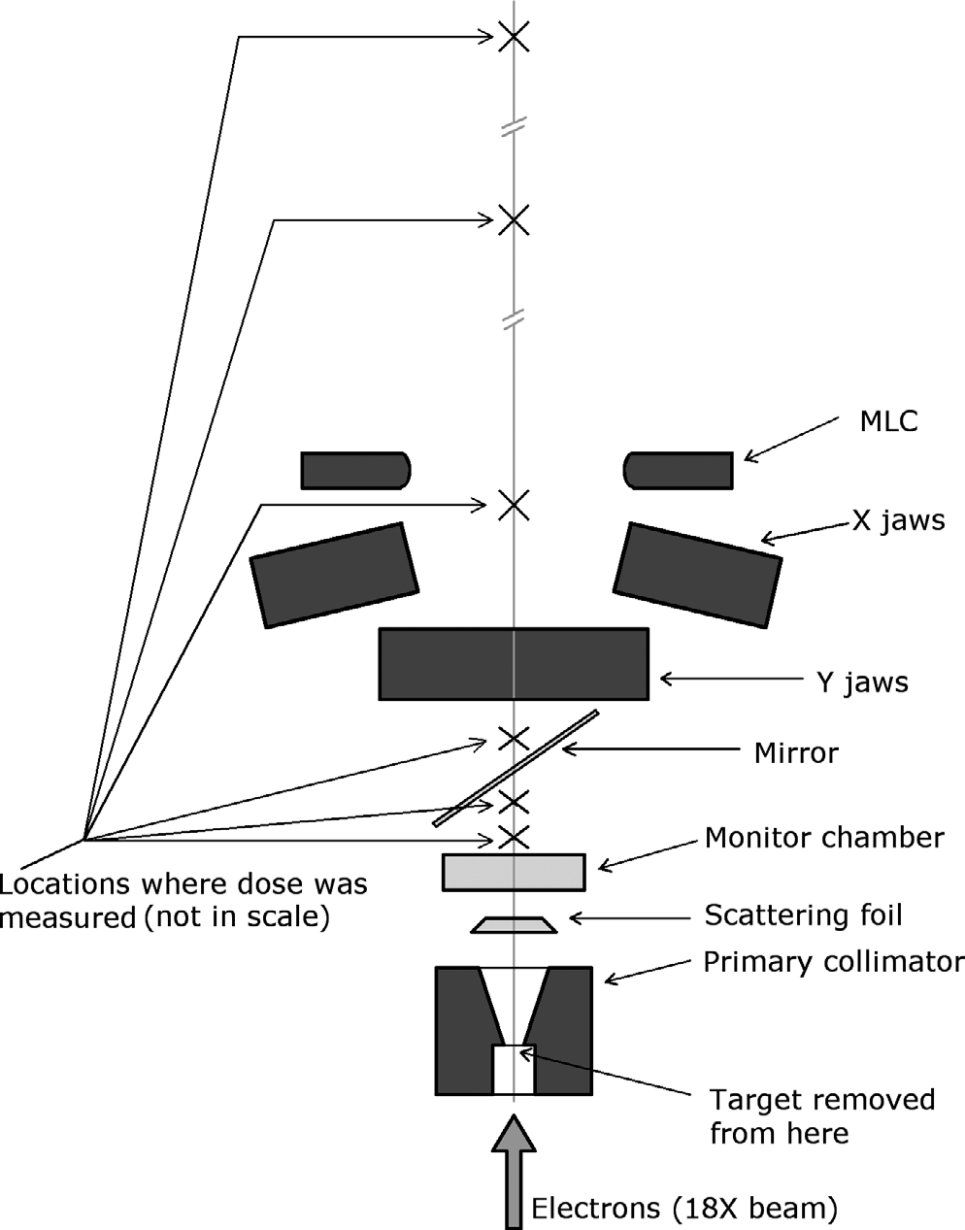
A diagram of the components in the linac head in the configuration used to deliver FLASH electron beams. The locations where the dose was measured are also shown (not in scale).

A clinically decommissioned Varian iX linac was used in the experiments. The diagram of the system is shown in Fig. 1, together with the locations (not in scale) where the dose was measured. A FLASH 18MeV electron beam was created from an 18MV photon beam (600 MU/min unless noted otherwise) through retracting the target, and replacing the flattening filter with an electron scattering foil. The scattering foil was mounted on the carousel as in a conventional electron beam. While it is always an electron beam travelling through the waveguide for both photon and electron clinical beams, a clinical nominally photon beam was chosen to be modified instead of modifying a clinical electron beam. This allowed taking advantage of significantly higher beam current in the waveguide for a photon beam with a flattening filter (where the target efficiency combined with attenuation in the flattening filter result in fluence reduction) compared to a typical electron beam. The 18MV photon board was used. Most of the measurements reported herein were performed with the 9 MeV scattering foil, but a few measurements were done with the 16‐MeV foil. Unless stated otherwise, the subsequent paragraphs pertain to measurements with the 9‐MeV foil. The carousel with the scattering foils and the flattening filter, and the target were prevented from moving to the default locations by re‐routing the pneumatic system that controls the movement of the carousel and of the target. Servo control of the dose and of the steering was disabled as the internal monitor chamber was expected to provide signal incompatible with the linac electronic circuits. The beam was tuned using a standard linac maintenance protocol (Varian, C‐Series Clinac® High Energy Technical Maintenance 2 Lab Guide, Rev. E).

The dose to be delivered was programmed by entering the number of MUs at the operator console in the service mode. This method is different from the method employed by Schüler et al., where a microcontroller connected to the linac gating system was used to terminate the beam.^7^

### 2.B Dosimetry

Three dosimeters were used: Gafchromic EBT‐XD film, OSL and cc13 ion chamber (IBA Dosimetry, Schwarzenbruck, Germany). EBT‐XD film was employed instead of more commonly used EBT3. Much lower sensitivity compared to EBT3 allowed achieving higher accuracy in measurements of high doses encountered in our FLASH beam measurements, where the optical density approaches saturation (for EBT3), and the ratio of the optical density to its uncertainty becomes unfavorable. In our experiments, the film was used following dose calibration in solid water in a conventional (non‐FLASH) 16‐MeV electron beam. All film pieces, including the film calibration pieces, were scanned with Epson Perfection V700 PHOTO flatbed scanner (Epson, Long Beach, CA, USA), and the red channel was used in the analysis. The film pieces were placed at the central axis of the scanner, that is, at the line across the light source of the scanner and passing through the middle of the light source, and in the same orientation of the film relative to the light source of the scanner.^18^, ^19^ Placement of the film at or near the optical central axis eliminated the need to correct nonuniformity of the dose measurement, which depends only on the distance from the central axis, and not on the location along the axis. The film was always scanned 1 hr after the irradiation to eliminate the error associated with time‐dependent changes of the optical density of the irradiated film following the irradiation. Film optical density was converted to dose using in‐house written software.

The OSL detectors were also calibrated in a conventional electrons beam and using solid water. During the subsequent experiments, both the film and the OSL were sandwiched between layers of superflab bolus (1.2 g/cc density, 2 cm thick upstream, 1 cm thick downstream) to keep the measurements in the flat portion of the PDD. Non‐water like bolus was used as there was not enough room to place slabs of solid water inside the linac head, but the small difference in the density from the density of water has little impact on our data: only about −3% dose difference, which is small compared to other uncertainties.

Out of the three detectors employed herein, only an ion chamber can be used in vivo during irradiations of radiobiological samples, as both film and OSL require post‐processing. The chamber was placed across the beam central axis (CAX) in a wax phantom (0.9 g/cc density, 6.6 cm diameter across the beam). Non‐water like medium was also chosen for practical reasons, and the errors caused by such choice are small, about 1%, compared to other uncertainties in this work. Even though the chamber was calibrated using a standard protocol (in a conventional electron beam at the reference conditions), the charge collection efficiency is known to decrease with an increase of the dose rate,^16^ and this effect will be investigated in the subsequent sections.

The dose rate was computed as the ratio of the dose measured with film to the irradiation time. The total time of irradiation was established by counting the video frames of Cherenkov glow filmed with a video camera. Cherenkov radiation coincided with the beam‐on, and in this experiment, the bluish glow was emitted when the MeV electrons from the FLASH beam were passing through the acrylic support plate and the semi‐transparent build‐up phantom (superflab and wax). iPhone (Apple Inc., Cupertino, CA, USA) recording at 240 fps was used as the video camera. The uncertainty of this method is 1 video frame, which corresponds to about 4 ms. The film was irradiated concurrently with video recording of the Cherenkov glow.

The instantaneous dose rate (during a linac pulse) was obtained from the average dose rate through dividing by the ratio of the pulse width (3.0 µs) to the time period between pulses (5.5 ms). These periods were measured with an oscilloscope for 18‐MV clinical photon beam operating at 600 MU/min, and they are same as in our FLASH electron beam. The corresponding dose‐per‐pulse (*DPP*) was computed from the average dose rate (Gy/s) by dividing by the number of pulses per second (the inverse of 5.5 ms pulse‐separation time).

The beam energy was verified by measuring the ratio of the FLASH‐mode PDDs at the depth of 7.0 cm and 3.5 cm (SSD = 100 cm, 10 cm × 10 cm), using optically stimulated luminescence (OSL) dosimeters (Landauer, Glenwood, IL, USA) sandwiched between slabs of solid water, and interpolating between the known ratios of conventional electron beams. This method was chosen over the more conventional measurement of R50, because it is quicker, as it does not require setup of a water tank system to collect the full PDD curve.

As a surrogate for *in vivo* animal radiobiology studies, a two‐piece phantom of a mouse was 3D‐printed (using PLA filament, 1.3 g/cc density) based on a CT scan of a mouse. EBT‐XD film was sandwiched in the coronal plane between the two halves of the phantom, and placed such that the film was orthogonal to the CAX.

### 2.C Efficiency of an ionization chamber

The efficiency *f* of charge collection by an ion chamber is defined as the ratio of the dose reported by the chamber to the actual dose. The former is the ionization charge converted to the dose using a calibration in a conventional beam, while the dose concurrently measured with film was used as the latter.

Four models of ion recombination were applied to model our combined measurements of the chamber efficiency in the FLASH beams (18 MV with 9‐MeV or 16‐MeV scattering foils) and in the conventional 16‐MeV electron beam:

- The conventional two‐voltage correction for pulsed beams with measurements done at 150 V and 300 V chamber bias.^16^
- Boag classical model of recombination^16^ derived for parallel‐plate chambers:

(1) $$f\;=\;\frac{\;1}{u}\;\ln\;\left(1+u\right)$$

where *u* = *k DPP*, and *k* depends on the gas in the chamber, the bias, and the distance between the electrodes. We are not aware of a similar model developed specifically for cylindrical chambers like cc13. A regression algorithm was used here with the factor *k* being the fitted parameter, while *DPP* was the independent variable.

c) and d) Ion recombination in the presence of free electrons (electrons liberated by an ionizing particle of the measured beam, which remain free prior to reaching the collecting electrode), also for parallel‐plate chambers:

(2) $$f\;=\;\frac{1}{u}\;\ln\;\left(1+\frac{e^{\mathrm{pu}}‐1}{p}\right)$$

and

(3) $$f\;=\;p+\frac{1}{u}\;\ln\;\left(1+\left(1‐p\right)u\right)$$

where the free‐electron fraction *p* is independent on the *DPP*.^17^ In addition to *k*, we used *p* as the fitted parameter. Equations (2) and (3) were originally derived assuming different charge distributions in the chamber.

The fitting weights were set as the inverse of the squared ordinate in order to assign equal percent uncertainty to all data points, in spite of the ordinate varying by almost two orders of magnitude. Neither the temperature and the pressure variations, nor the bias polarity, were corrected for, but these are second‐order corrections compared to the corrections required for FLASH dose rates.

## 3 RESULTS

### 3.A Beam energy

**Fig. 2:**
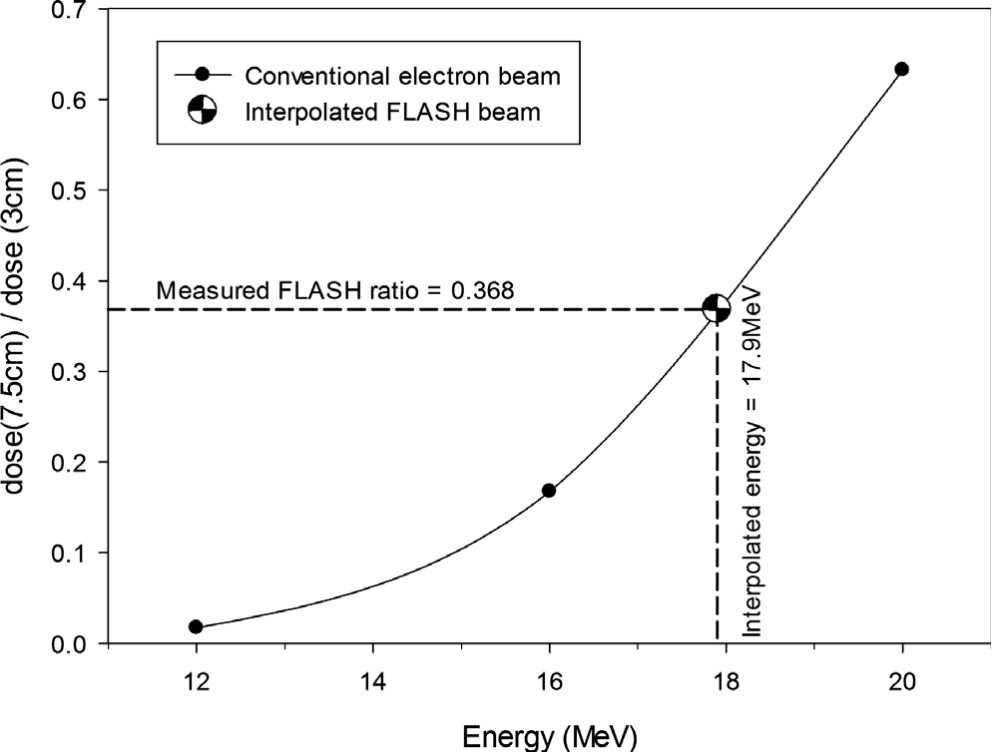
Verification of the FLASH‐beam energy through interpolation of the depth‐dose ratios of conventional electron beams (16 MeV scattering foil).

The beam energy was measured to confirm the energy of the electrons in the waveguide was set correctly, that is, 18 MV beam was modified, not 6 MV or other beam. Herein it was measured when the 16‐MeV scattering foil was in place. Verification of the energy of our FLASH electron beam is illustrated in Fig. 2. Interpolation of the ratio of the PDD at 7.5 cm and at 3 cm depths yielded the beam energy of 17.9 MeV. This is consistent with our expectation following modification of the 18‐MV photon beam after replacement of the target and the flattening filter with a scattering foil.

### 3.B Dose reproducibility and linearity with MU

**Fig. 3:**
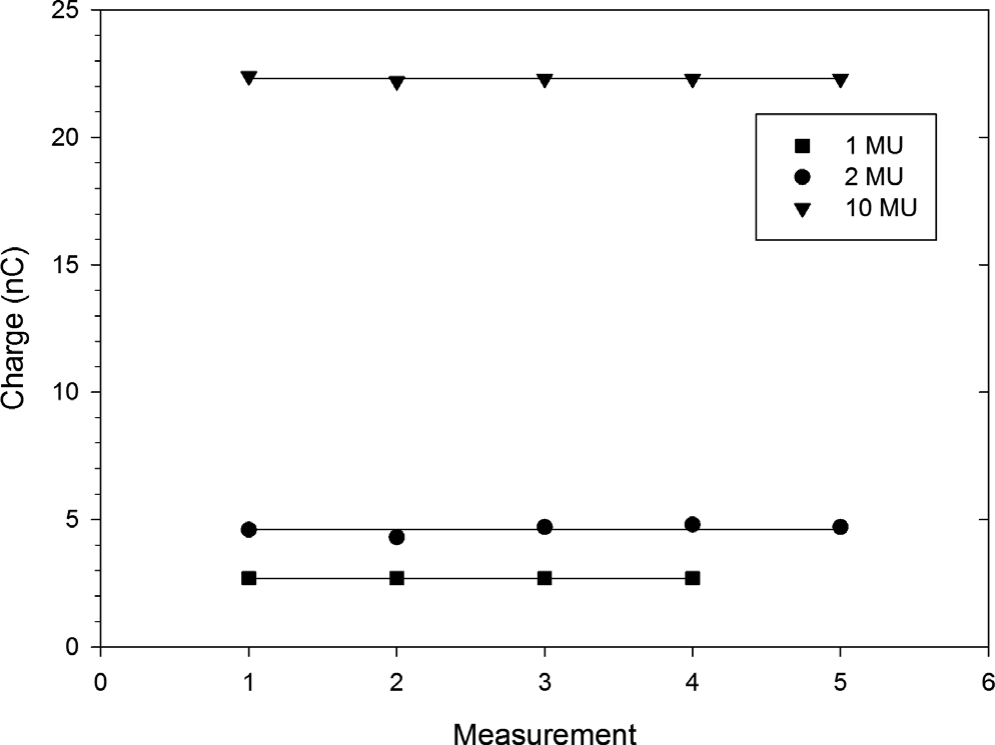
Reproducibility of the delivered dose with FLASH beam as measured with the cc13 chamber for various MUs. The lines indicate the corresponding averages.

Reproducibility of the delivered dose was assessed at different values of MU. Figure 3 shows the charge collected by the ion chamber mounted near the MLC (SAD = 49 cm). Stability of the delivered dose is satisfactory. The standard deviation (0.1 to 0.2 nC) appears to be approximately independent on the delivered dose, and can be considered an additive, not multiplicative, uncertainty. Naturally, the relative uncertainty improves with an increase of the dose. The smallest deliverable dose is 1 MU, which is a limitation of the iX linac model.

**Fig. 4:**
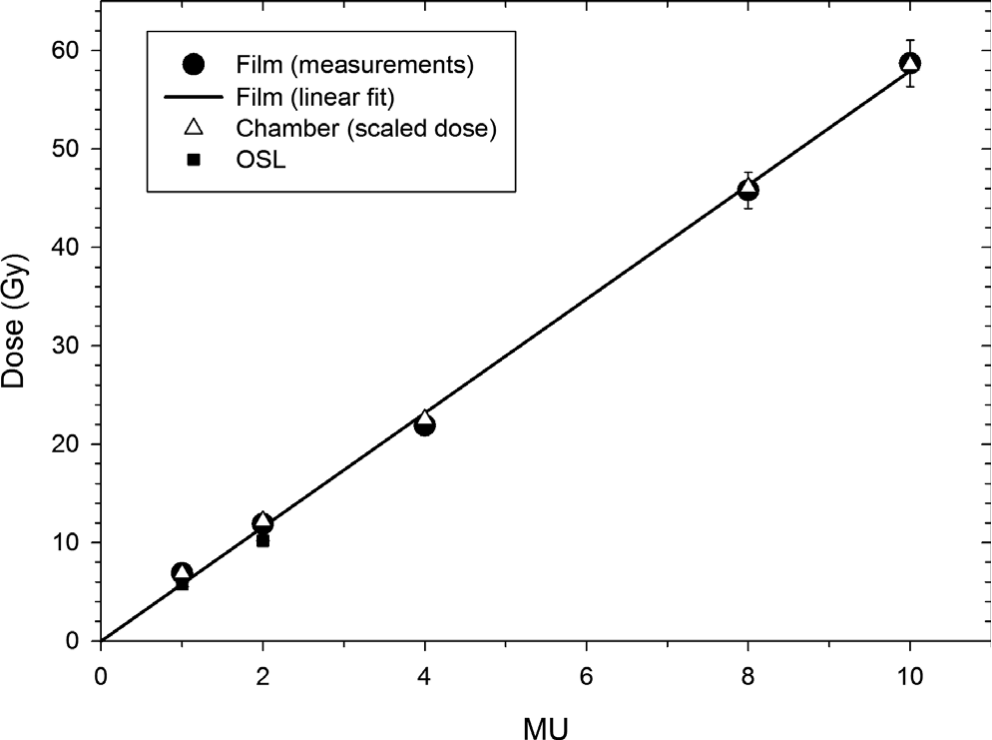
Dose vs. MU measured at SAD = 49 cm using film (absolute dose), cc13 chamber (relative dose), and OSL (absolute dose).

Dose linearity vs MU was confirmed by employing film, OSL, and the cc13 chamber, and is depicted in Fig. 4. The dose measured with film conforms to the linear fit with the intercept of 0 Gy. The dose measured with OSL is limited to small doses, and agrees with the dose measured with film. The charge measured with the chamber was scaled to match the film data at 10 MU, and should be considered relative dose only. The relative doses measured with the chamber agree with the film and the OSL data for all values of MU.

### 3.C Dose dependence on the repetition rate

**Fig. 5:**
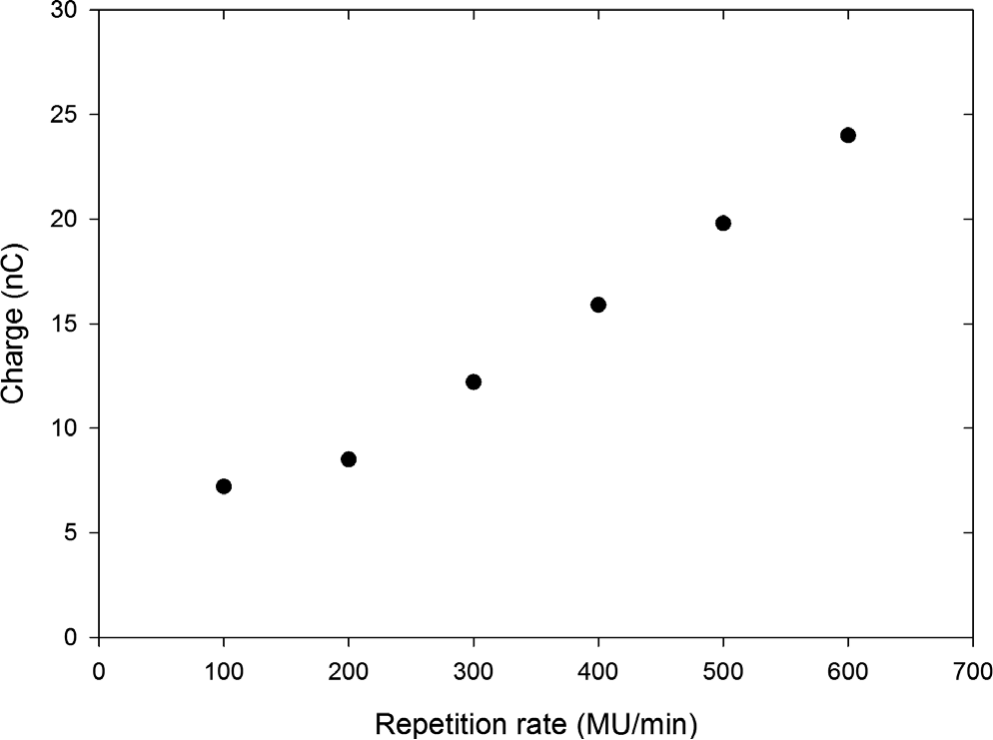
Dependence of the charge measured with the cc13 chamber on the repetition rate in the FLASH electron mode (16 MeV foil).

**Fig. 6:**
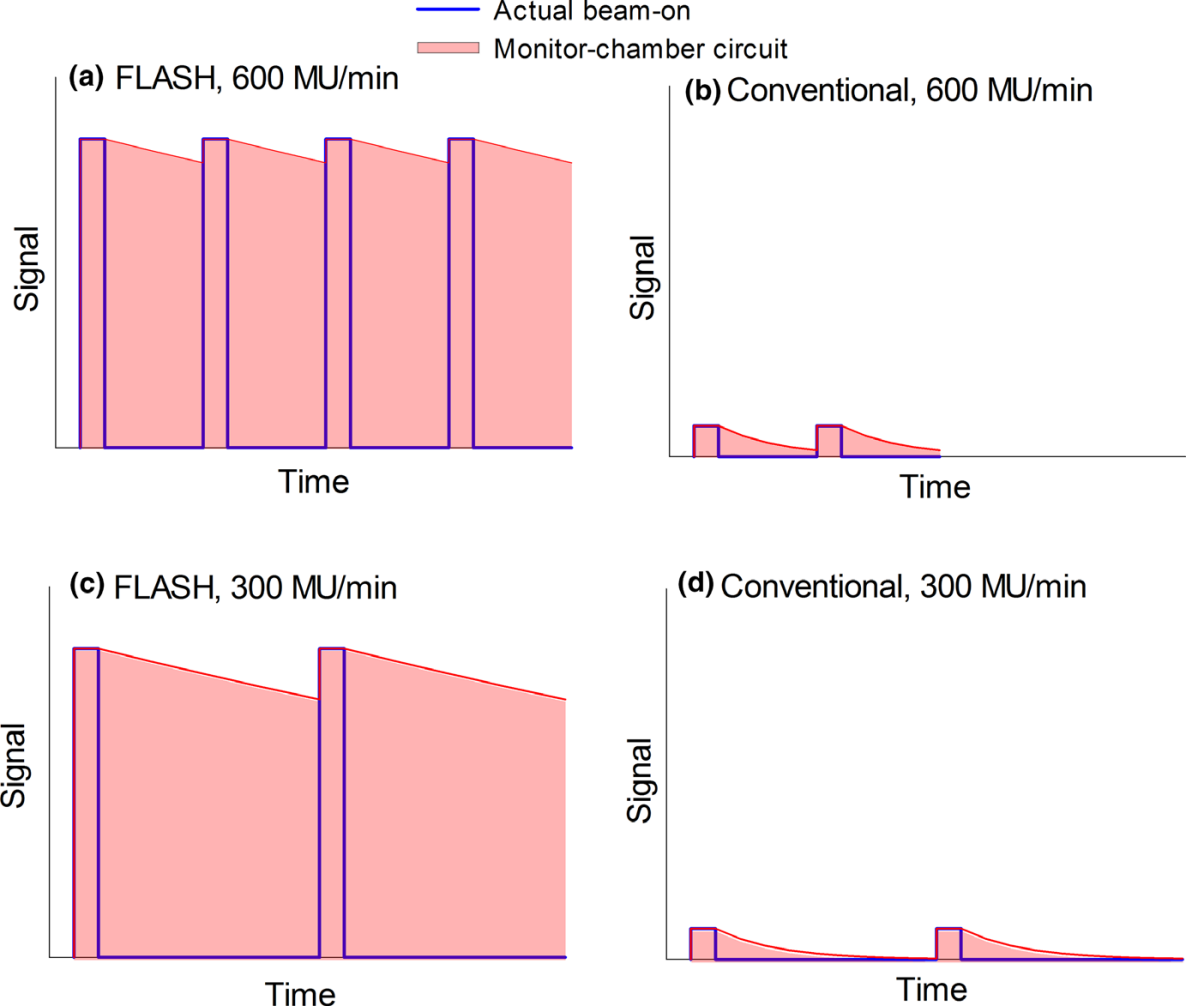
Proposed model explaining how the dose depends on the repetition rate in: (a) and (c) FLASH and in: (b) and (d) conventional beam.

The dependence of the charge measured with the cc13 chamber on the repetition rate is shown in Fig. 5. The data in this section were collected with the 16‐MeV instead of the 9‐MeV scattering foil due to reasons unrelated to this manuscript. The measured charge increases with the repetition rate in an approximately linear manner. This is dramatically different than a conventional electron beam, where the measured charge is independent of the repetition rate. We hypothesize the relationship observed in the FLASH beam is due to the signal of the internal monitor chamber not being fully reset prior to the arrival of the next pulse. This is conceptually illustrated in Figs. 6(a) and 6(c) for 300 MU/min and 600 MU/min FLASH beams. Following a pulse (shown in blue), the signal at the internal monitor chamber (red) does not decay enough before arrival of the next pulse, that is, the signal remains approximately constant in the FLASH mode no matter how many pulses arrive per unit of time. The beam gets terminated when the time integral of the monitor chamber signal (the red area under the red plot) reaches the preset value. The beam cutoff time is approximately same for both repetition rates. As twice as many pulses are delivered per MU (and per unit of time) at 600 MU/min compared to 300 MU/min, the total charge recorded on the external chamber is doubled for 600 MU/min compared to 300 MU/min, and this is what is seen in Fig. 5. In the conventional mode, Figs. 6(b) and 6(d), where the amplitude of the signal at the circuit is much lower, the signal gets reset to zero before the arrival of the next pulse, and the relative contribution of the signal decay to the time integral is small compared to the contribution of the actual beam‐on pulses. The beam gets terminated when the time integral reaches the preset value, and the same number of beam pulses are delivered per MU no matter what the repetition rate is. Consequently, the delivered dose is independent on the repetition rate in the conventional beam. The charge collected at 100 MU/min (Fig. 5) appears to deviate slightly from the linear trend seen at higher MU/min, that is, the charge is almost the same for 100 MU/min and 200 MU/min. At such low repetition rate, the separation between the pulses is large enough to allow the signal on the monitor chamber circuit to decay before arrival of the next pulse, and the system acts more like with a conventional beam where the charge is independent of the repetition rate.

### 3.D Beam profiles and field size dependence on SAD

**Fig. 7:**
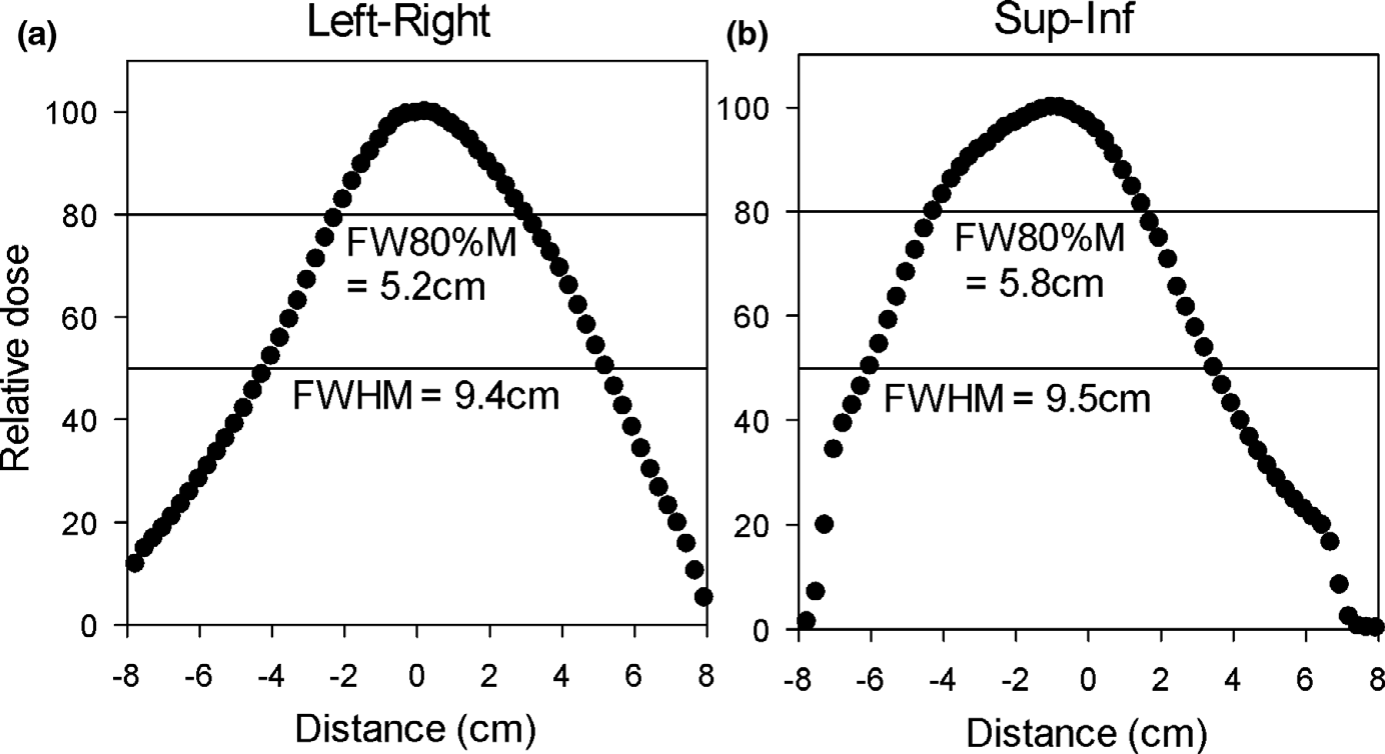
The dose profiles at SAD = 49 cm in the FLASH beam.

An example of FLASH‐beam profiles measured with film is shown in Fig. 7. The profiles were collected at SAD = 49 cm. Both inline and crossline profiles are not flat because the scattering foil that was used (9 MeV) was too thin to provide effective scattering of the 18 MeV incident beam. The shape of the inline and the crossline profiles is similar. The centroid is slightly shifted from the CAX, which is likely caused by disabling the steering servo without adjusting the steering manually. The FW80%M is about 5.5 cm, and this number limits the area usable in radiobiological experiments.

**Fig. 8:**
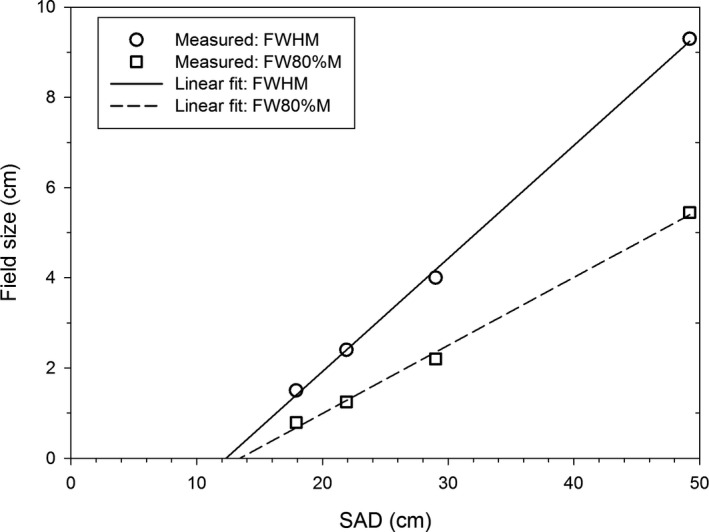
The dependence of the field size on the distance from the nominal beam center.

Dependence of the field size on the SAD is plotted in Fig. 8. We investigated locations downstream from the internal monitor chamber. The ratio of the FWHM and the effective SAD follows the beam divergence (the theorem of similar triangles). The fitted value of the effective beam center (the intercept of the linear fit at the field size equal 0 cm) is 12.5 ± 0.5 cm downstream from the nominal beam center. The data of the FW80%M also follows the theorem of similar triangles, with a very similar value of the effective center (13.5 ± 0.8 cm). These values of the effective beam center coincide with the location of the scattering foil, which is mounted about 12.5 cm from the nominal beam center. The effective beam origin is not at the target location, because the target is removed in our FLASH beam, and the angular beam scattering occurs at the scattering foil. It should be noted that the angle of the beam divergence is not set by the opening of the primary collimator (which is upstream of the effective beam origin), but by thickness of the scattering foil.

### 3.E Two‐dimensional dose distribution in a mouse phantom

**Fig. 9:**
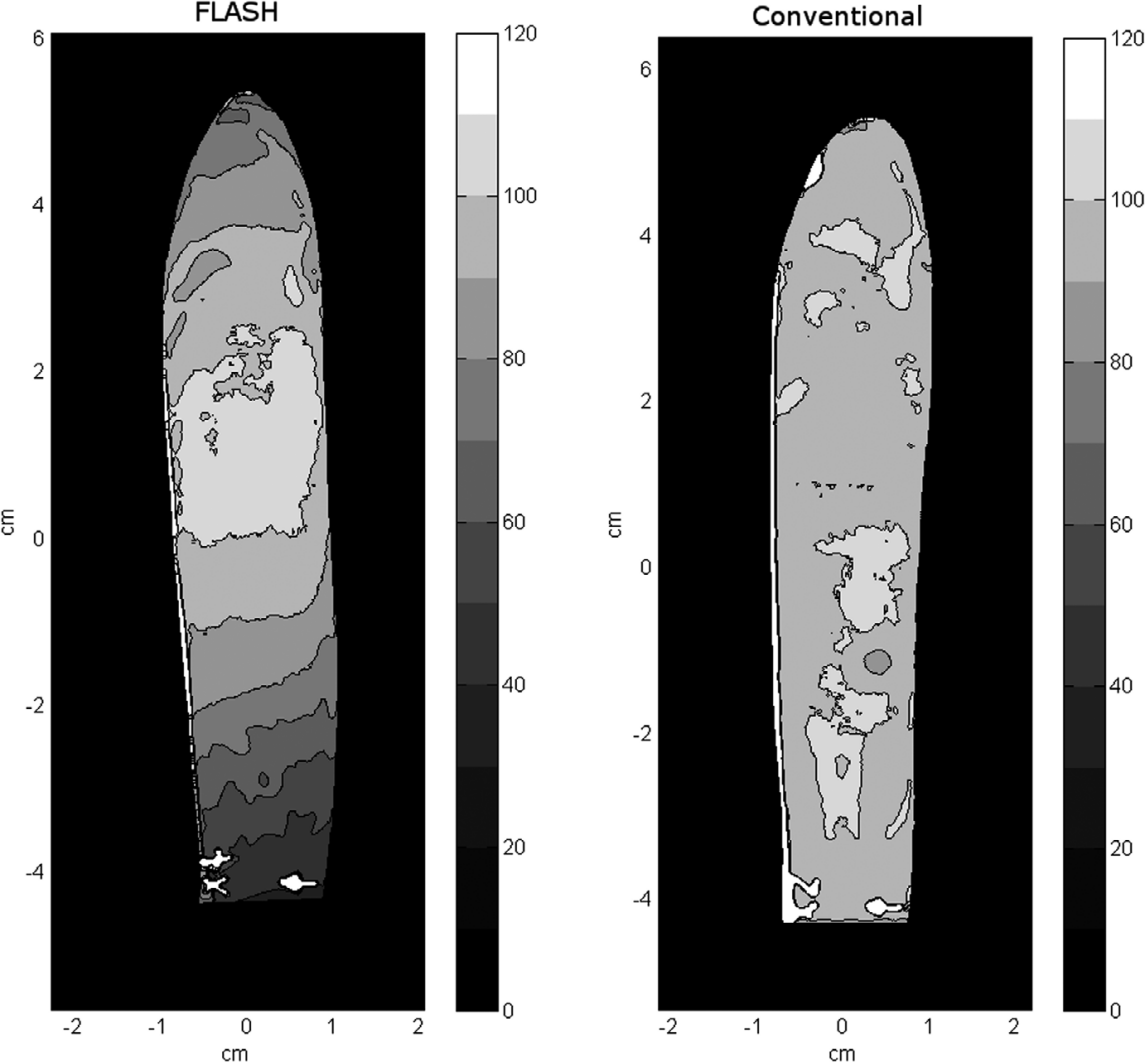
The relative dose distribution in the coronal plane of the 3D mouse phantom irradiated with the FLASH electron beam (18‐MeV beam, 9‐MeV foil, SAD = 49 cm) and the conventional electron beam (16 MeV, at the isocenter).

The two‐dimensional (2D) maps of the relative dose distribution in the mouse phantom are presented in Fig. 9 for both the conventional and the FLASH beams. The high‐dose like spots at the bottom are the film labels. While the dose distribution of the conventional beam is very uniform, it is not so for the FLASH beam. The dose distribution in the FLASH case is noticeably peaked around the CAX. The dose distribution near the edges of the phantom in the left‐right direction is rather flat, with no dose buildup near the edges. The area closer than about 2 mm from the edge of the film should not be used to draw conclusions about the dose distribution as it may be affected by light scattering during film scanning. The absence of lateral dose buildup may be useful in designing radiobiological experiments.

### 3.F The dose rate vs. MU

**Fig. 10:**
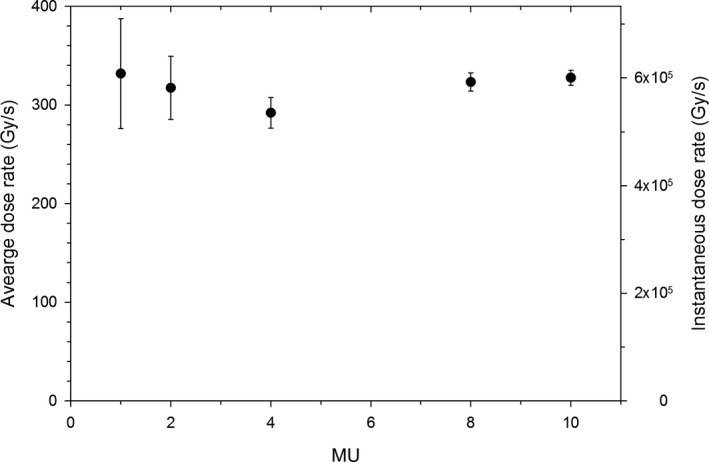
The dose rate measured with film (at SAD = 49 cm) computed using the time obtained from counting the video frames of Cherenkov glow.

The dose rate (the average and the instantaneous value during a linac pulse) obtained from film irradiated during recording of the Cherenkov glow is plotted in Fig. 10. The dose rate appears not to depend on the number of delivered MUs, even at 1 or 2 MU. This is the same behavior we see in conventional linac beams. Schüler et al.,^7^ reported the values of the dose per pulse observed in a FLASH linac controlled with a microcontroller were smaller during the first few pulses, and this would affect the dose delivered at small MUs, for example, 1 MU. It is difficult to state whether the same happens when the dose is prescribed by entering the MUs through the console due to considerable error bar in our experiment for 1 MU (the uncertainty of measuring the time of irradiation is equal to one frame of the video).

### 3.G The dose rate vs. SAD

**Fig. 11:**
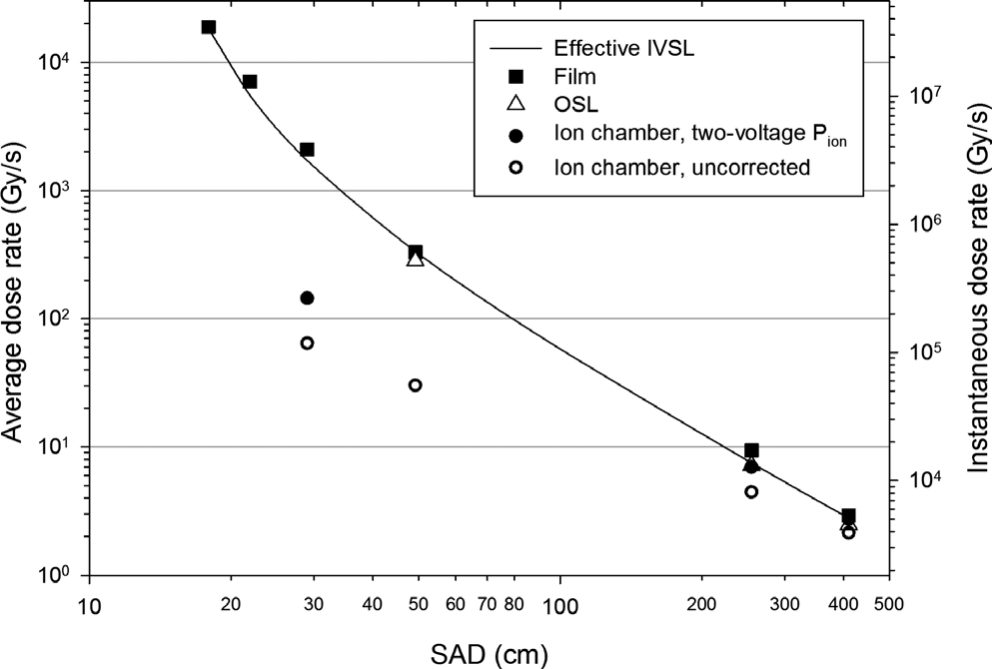
The dose rate at various distances from the nominal beam center for the FLASH electron beam. The measurements (film, OSL, and cc13 chamber) are shown together with the effective IVSL model (scaled to the film data at SAD = 408 cm).

Dose‐rate dependence on the distance from the nominal beam center is plotted in Fig. 11 for various dosimeters: film, OSL, and cc13 chamber. Both the average and the during‐the‐pulse instantaneous dose rate are shown. Also plotted is the theoretical model, which is the inverse square law (IVSL) with the effective beam center set to 13.0 cm downstream from the nominal beam center. This value was taken from the x axis intercept in the plot of the field size divergence vs. the distance from the nominal beam center, as reported in Fig. 8. The IVSL model was scaled to match the film data at the largest distance (408 cm), for which verification is not required, because the dose rate is comparable to the value employed during film calibration with a conventional electron beam. The film data agrees very well with the IVSL model, even at the shortest SAD. This is remarkable considering the dose rate changes about four orders of magnitude. This agreement with the model demonstrates independence of the dose measured with the EBT‐XD film on the dose rate up to about 2 × 10^4^ Gy/s.

The OSL data in Fig. 11 agrees with the film data and the IVSL model. We did not collect OSL data at dose rates exceeding 280 Gy/s due to excessive values of the ratio of the OSL reading to the statistical uncertainty.

Figure 11 contains both the as‐measured (uncorrected) chamber data and the chamber data corrected for ion recombination using the conventional two‐voltage method. The uncorrected chamber data agrees with the film data only at the large SAD (>2.5 m). The two‐voltage P_ion_ correction is not adequate for FLASH dose rates: at SAD = 30 cm it under‐reports by a factor of about 10. While more data should be ideally collected to reject the two‐voltage model of P_ion_, inapplicability of this method to FLASH dose rates is known considering the approximations used in the derivation are valid only at low dose rates.^12^, ^17^

### 3.H Chamber efficiency

**Fig. 12:**
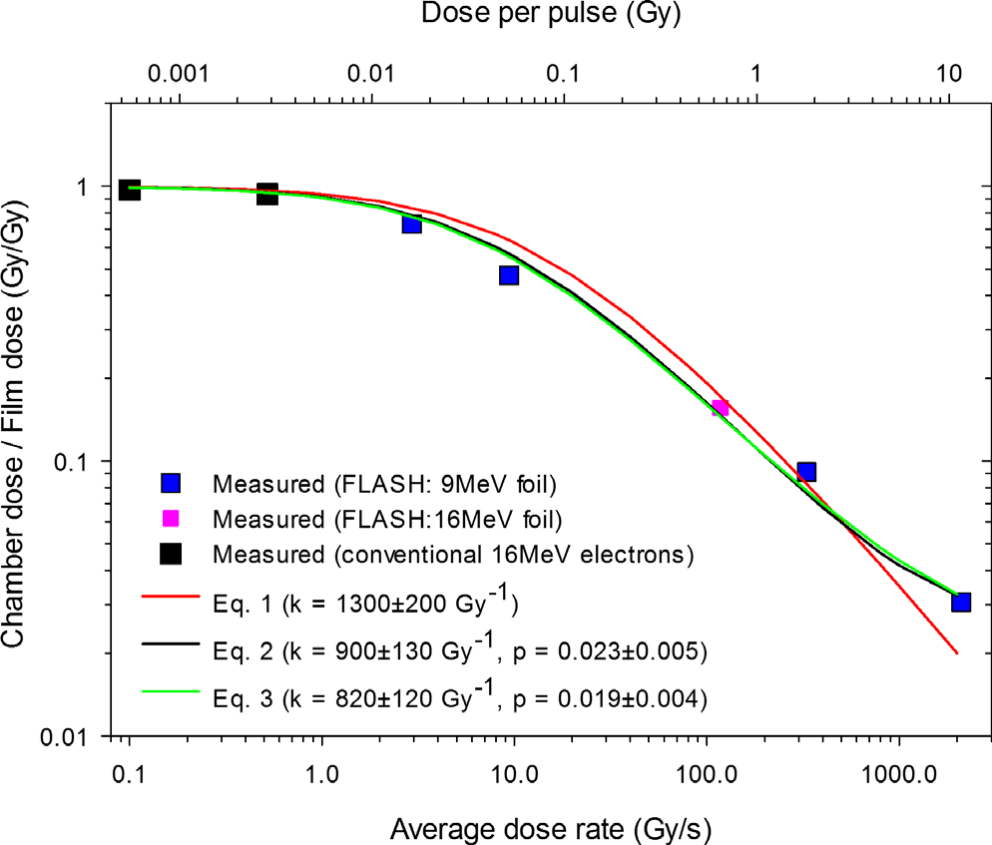
Measured (symbols) and modeled (lines) efficiency of the cc13 chamber at various dose rates (i.e. at various dose‐per‐pulse) for the repetition rate of 600 MU/min. The FLASH data measured at various distances from the beam center is combined with the data measured with the conventional 16‐MeV electron beam.

The measured chamber efficiencies for various dose rates are plotted in Fig. 12 for the FLASH (9 MeV and 16 MeV scattering foils) and the conventional electron beam measurements. The dose rate was varied by changing the SAD or beam configuration (conventional or FLASH with 9 MeV or 16 MeV scattering foil). The efficiency of one indicates no need to apply any corrections to the chamber data. The ordinate is plotted in the logarithmic scale to emphasize the practical need of obtaining similar percent uncertainty at any value of the efficiency. The two‐voltage model is entirely inadequate in the FLASH dose rates, and is not shown here for clarity. The classical Boag model^16^ (Equation 1) works well for the low and the medium values of the dose rates, but it underestimates for the highest dose rate significantly (by a factor of 2). One could adjust the value of the parameter *k* for a better match at the highest dose rate, but agreement at intermediate dose rates would be compromised (not shown for clarity). Much better agreement, especially at the highest value of the dose rate, is observed with the models which take into account the presence of free electrons (Equations 2 and 3). The fit quality of the two models with a free‐electron fraction is practically identical, although the values of the fitted parameters *k* and *p* differ slightly.

## 4 DISCUSSION

### 4.A Evaluation of the FLASH beam

Our measurements demonstrate the desired dose to be delivered by a linac converted to FLASH electron mode can be programmed by entering the MUs through the console, without use of a microcontroller. The only discovered limitation is the minimum deliverable dose (i.e., the dose corresponding to 1 MU), but this might not be an issue with Varian TrueBeam (or other) linac, which may deliver fractional MU. For doses corresponding to less than 1 MU, linac performance should be verified. After all, beam instability for beam‐on sequences utilizing a low number of linac pulses was reported.^7^

The set of measurements performed herein should provide confidence in the reliability of dose delivered in the FLASH mode prior to the start of a radiobiological experiment. The highest observed dose rate was 2 × 10^4^ Gy/s with the field size (FW80%M) of 1.2 cm, about 1 cm downstream from the monitor chamber (on top of 1‐cm thick buildup layer of superflab) with the mirror removed. This value of the dose rate exceeds the definition of FLASH dose rates by a factor of 500. The value of 2 × 10^4^ Gy/s is considerably higher than the value of 0.9 × 10^3^ Gy/s reported by Schüler et al.,^7^ or 1 × 10^3^ Gy/s reported by Lempart et al.^8^ The difference could be due to internal limits of tuning the beam utilizing a photon‐beam board (this work) compared to tuning with an electron‐beam board (Schüler et al.^7^). While some might consider the field size (FW80%M) of 1.2 cm too small for practical use, it is not small relative to cell colonies or the size of a tumor in a small animal. There is a reasonable amount of space in that area to accommodate purposely designed sample holder. For even higher dose rates, it might be possible to mount samples on the carousel in the holder of the scattering foils, but the protocol for changing the samples would be more complex, and the number of MUs to enter would be affected due to the monitor chamber being partially blocked by the irradiated sample.

It should be noted that the beam centroid might be shifted from the nominal central axis (CAX) when the steering servo is disabled, and care should be taken to adjust the steering with the servo off. Similarly, the dose rate may fluctuate due to lack of dose rate servoing. It is recommended to monitor the dose rate during all sequences. This can be done by utilizing an external ion chamber, ideally with the chamber efficiency correction described in this manuscript (Equation 4 introduced in Section 4.C). Servoing of the dose could be restored if the corrected signal of the external chamber was employed instead of the internal chamber, but such modification is beyond the scope of this paper.

### 4.B Gafchromic film and OSL

We demonstrated Gafchromic EBT‐XD film can be used without any dose‐rate corrections for dose rates encountered in FLASH experiments, at least up to 2 × 10^4^ Gy/s. This was concluded upon observing agreement between the doses measured with film at various distances from the scattering foil and the doses computed using the effective inverse square law. In the absence of scattering on linac components for a rather narrow beam (for the 9 MeV scattering foil), other than the internal monitor chamber with thin beam‐through windows, and for distances from the effective beam source considerably larger than the beam size at the source, the inverse square law, which is a fundamental property of space, may serve as a reliable gold standard. This method of confirming dose‐rate independence of a detector (film here) is different than using a Faraday cup or monitoring beam current,^10^, ^11^ and does not require additional instruments.

Similarly to the film data, the OSL measurements presented in Figure 11 agree with the inverse‐square model. This demonstrates independence of the OSL readings on the dose rate up to about 280 Gy/s. The dose rates encountered in OSL measurements reported in Figs. 2 and 4 do not exceed this value.

### 4.C Use of cc13 ionization chamber in ultra‐high dose rates

It should be noted that the chamber efficiency in the recombination models applied herein depends on the dose per pulse, and care should be taken when using the dependence on the average (and during‐the‐pulse) dose rate. The conversion factor between the *DPP* and the average dose rate would be different for different number of pulses per second, for example, if the repetition rate was different than 600 MU/s or even if different beam energy was used (the timing sequence of a linac depends on the beam energy). This conversion factor is independent of the distance from the nominal beam center and the choice of the scattering foil, because these factors do not affect the timing sequence of the beam.

While a reasonable agreement between the measured and the modeled chamber efficiency was achieved in this work, the discrepancies exceed clinically used tolerance of 2%. The average absolute value of the discrepancy across the entire investigated range of dose rates is 7% with the standard deviation of 5%. This may be due to inability of the models tested in this work to adequately model the complex events of ion or electron recombination. In particular, the tested models were derived for parallel‐plate chambers, while the cc13 is a Farmer‐type chamber. The values of the free‐electron fraction fitted to the measurements in this work are considerably smaller than the values computed by Boag et al.^17^ or measured by Hochhauser et al.,^20^ but this again might be due to different geometry of the cc13 chamber. Nevertheless, both parameters *p* and *k* should be independent of the *DPP* even in the spherical/cylindrical geometry of the cc13 chamber, and using them to describe the dependence on the dose rate (and consequently on the *DPP*) has merit. Considering substantial uncertainties in measurements or calculations of the free‐electron fraction, we believe fitting the parameters of the models is a good method to model the dependence of the chamber efficiency on the dose rate. Petersson et al.^12^ performed similar modeling of chamber efficiency, but they evaluated a parallel‐plate chamber (Advanced Markus) instead, which is different from the cc13 cylindrical chamber evaluated here. Also, the range of efficiencies tested herein (down to 0.03) is considerably broader than tested by Petersson et al.^12^ Further investigation would be advisable, but is beyond the scope of this article.

In the sections dedicated to measurements of the fundamental FLASH beam properties, the cc13 chamber was used as a relative dosimeter operating at the same values of Gy / s, where the chamber efficiency remains constant. Establishing the chamber‐efficiency correction was meant for use in future experiments. The relationship describing the dependence of the chamber efficiency on the dose rate cannot be used directly to measure the dose, because the dose rate is not necessarily known (unless we assume constancy with respect to previously characterized conditions). Nevertheless, the dose rate *r* is linked to the value of the chamber efficiency *f* and the uncorrected dose measured with the chamber *d_m_* as:

(4) $$r\;=\;d_{m}\;/\;\left(f\tau \right)$$

where the time of irradiation τ can be established by multiplying the *MU* by *MU*‐to‐*time* conversion factor established for the beam e.g. from analysis of video of Cherenkov glow. Incidentally, this conversion factor is independent of the SAD. Inserting Equation 4 into the relationship between the chamber efficiency and the dose rate (as seen in Fig. 12) leads to an analytically unsolvable equation where both sides depend on *f*, but such an equation can be solved numerically in real time. It should be noted that any discrepancies in modeling of the chamber efficiency will affect accuracy of deciphering the value of the chamber efficiency using this procedure, and for example, a logistic model^12^ or even simple spline interpolation might provide adequate results.

## 5 CONCLUSION

It was demonstrated the dose in a FLASH electron beam (obtained by replacing the target in a nominally photon beam with an electron scattering foil) can be prescribed by entering the MUs through the console, similarly to the way a conventional linac is controlled. A set of basic dosimetric tests was performed, and, with exception of the dependence on the repetition rate, the results exhibited similar trends as in the conventional beams. Applicability of the EBT‐XD film to dosimetry in FLASH mode was shown. A model of ion recombination in cc13 ionization chamber was identified to eliminate dose‐rate dependence from dose measurements.

## AUTHOR CONTRIBUTIONS

Stanislaw Szpala wrote the manuscript, designed and performed most of the measurements. Vicky Huang designed and 3D printed the mouse phantom, and performed the subsequent measurements. Yingli Zhao participated is selected measurements. Alastair Kyle and Andrew Minchinton assisted with measurements utilizing Cherenkov radiation. Tania Karan participated in the initial setup of the experiment. Kirpal Kohli initiated and supervised the project.

## CONFLICT OF INTEREST

No conflict of interest.

## Data Availability

The data that support the findings of this study are available from the corresponding author upon reasonable request.
